# Antifungal Activity of ToAP2D Peptide Against *Sporothrix globosa*

**DOI:** 10.3389/fbioe.2021.761518

**Published:** 2021-10-21

**Authors:** Tianyi Yan, Fuqiu Li, Jinran Li, Feng Chen

**Affiliations:** ^1^ Department of Dermatology, Second Hospital of Jilin University, Changchun, China; ^2^ Department of Dermatology, China-Japan Union Hospital of Jilin University, Changchun, China

**Keywords:** antimicrobial peptide, design and modification, apoptosis, *Sporothrix globosa*, anti-fungal activity

## Abstract

Improving clinical efficacy and reducing treatment time have been the focus of sporotrichosis therapy. Antimicrobial peptides ToAP2A, ToAP2C, and ToAP2D were synthesized on the basis of ToAP2 (AP02759), a peptide derived from the antimicrobial peptide database by the database filtering technology, and their physicochemical characteristics were analyzed. Compared with template peptide ToAP2, the modified peptides had much shorter length, lower molecular weight but significantly greater stability, which in return resulted in increases in the aliphatic index, hydrophilicity, and protein binding ability. Here, we show that the three derived peptides inhibit the growth of *Sporothrix globosa*, among which ToAP2D had the strongest anti-fungal activity. ToAP2D showed good serum stability without acute toxicity. The ToAP2D treatment inhibited the growth of *S. globosa* and enhanced apoptosis, which was evidenced by the upregulation of apoptosis-related protein caspase-3. The scanning electron microscopy analysis revealed deformation and rupture of *S. globosa*. The levels of mitochondrial membrane potential were decreased and that of the reactive oxygen species (ROS) were increased in *S. globosa* upon ToAP2D treatment. Moreover, ToAP2D activated metacaspase. In the *in vivo* study, we further demonstrated that ToAP2D inhibited the *S. globosa* infection of mice footpads, and its efficiency was nearly comparable to itraconazole. In summary, our results suggest that antimicrobial peptide ToAP2D has the potential for sporotrichosis therapy.

## Introduction

Sporotrichosis is a subcutaneous mycotic infection, which causes localized cutaneous and even severe systemic disseminated infections, and its global incidence increases every year (Etchecopaz et al., 2021). *Sporothrix globosa* is the predominant etiologic agent that brings about sporotrichosis in East Asia, notably in northeast China (Yao et al., 2020). An epidemiologic study found that approximately 1.6 million people die from fungal infections in the world each year (Struyfs et al., 2021a), and a global increase in sporotrichosis incidence occurs every year, especially in the tropical and subtropical regions (Chakrabarti et al., 2015). Current therapies to treat sporotrichosis are very limited, which mainly include antifungal drugs, photodynamic therapy, thermotherapy, cryotherapy, and surgery, and the effects are still far from satisfactory (Honse et al., 2010; MF Matos et al., 2020; Soeiro Sampaio et al., 2020). A more efficient treatment for sporotrichosis is greatly desired.

Antimicrobial peptides (AMPs) are commonly found in natural organisms, and they have a wide range of functions, including anti-pathogens (Stariha and McCafferty, 2021; Chang et al., 2021; Kim et al., 2021; Sabia Junior et al., 2019), anti-cancer (Rubin and Qvit, 2020), and immune regulation (Sun et al., 2015). The current antimicrobial peptide database (http://aps.unmc.edu/AP/main.html) includes 1,210 types of peptides with antifungal activity. The specific mechanisms of antimicrobial peptides mainly include plasma membrane disruption and non-membranolytic cytotoxicity. The plasma membrane disruption pathway indicates that the positively charged antimicrobial peptides bind to the negatively charged membrane of the pathogens, destroying its integrity, leaking contents, and causing death (Guilhelmelli et al., 2013; Deslouches and Di, 2017; Mwangi et al., 2019). In addition, non-membranolytic cytotoxicity is also involved in antimicrobial activity, such as genetic material damage, immune regulation, and apoptosis (Wassing et al., 2015; Struyfs et al., 2021b). Unlike traditional antibiotics, antimicrobial peptides have many advantages such as quick killing effect and low tendency to induce resistance (Guilhelmelli et al., 2016; Xu et al., 2020; Jayawant et al., 2021). However, natural antimicrobial peptides are usually longer peptides with relatively low activity, high cytotoxicity, and strong antigenicity, and these drawbacks limit their applications (Kreling et al., 2016). Hence, modifying and tailoring the structure of AMPs that allow us to circumvent the limits may find more applications.

A previous study showed that scorpion venom–derived antimicrobial peptide ToAP2 (AP02759) could inhibit the growth of *Candida albicans* (Guilhelmelli et al., 2016). Moreover, ToAP2 has the characteristics of low hemolytic, low cytotoxic, and remarkable antifungal biofilm activity, suggesting that it had great potentials for the development of new antifungal drugs (Guilhelmelli et al., 2016). As the biological characteristics of *S. globosa* are very similar to *C. albicans*, we then use ToAP2structure as a template, through reasonable molecular design and modification, to study the inhibitory effect of antimicrobial peptides on sporotrichosis.

## Materials and Methods

### Materials

Antimicrobial peptides ToAP2A, ToAP2C, and ToAP2D were purchased from Chinapeptides Co. Ltd. (Suzhou, China). The Hoechst 33342–propidium iodide (PI) double-staining apoptosis-detection, ROS detection assay, and JC-1 kits were purchased from Beyotime Institute of Biotechnology (Jiangsu, China). The Cas-pACE FITC-VAD-FMK *in situ* marker was acquired from Promega Corporation (Fitchburg, WI, United States). All of these reagents were used directly without further treatment. Female BALB/c mice (8–10 weeks of age; body weight: 16–20 g) were purchased from Changchun Yisi Experimental Animal Technology Co. (JiLin, China) and used for all *in vivo* experiments.

### Antimicrobial Peptide Bioinformatic Analysis

On the basis of the antimicrobial peptide ToAP2 (AP02759), we designed antimicrobial peptides ToAP2A (GSKLIPGVMKLFSKK), ToAP2B(FFGTLF KLGSKLIPGV), ToAP2C(GAKLIPGVMKLFRKK), ToAP2D (GDKLIPGVMK LFRKK) by intercepting its effective fragments and substituting amino acids. Physical and chemical parameters of the newly designed peptides were analyzed using ExPASy Prot Param. The secondary structures of the peptides were constructed by HNN software of NPS@.

### *S. globosa* Preparation and Culture Conditions

*S. globosa* was collected from clinical patients diagnosed with cutaneous sporotrichosis at our hospital. All strains were identified as *S. globosa* using mycelial-to-yeast phase conversion culture and molecular identification methods. All isolates were grown on Sabouraud’s dextrose agar. After 7 days of culture, the fungal colonies were transferred to brainheart infusion broth and incubated in a rotary shaker (150 rpm/min) at 37°C for another 7 days. *S. globosa* was centrifuged and resuspended in PBS and diluted to 1.0 × 10^8^ cfu/ml.

### Antimicrobial Peptides ToAP2A, ToAP2C, and ToAP2D Against *S. globosa In Vitro*

The antifungal activity of ToAP2A, ToAP2C, and ToAP2D against *S. globosa* was analyzed using the inhibition zone test. *S. globosa* was collected and diluted to 1.0 × 10^8^ cfu/ml with a hemocytometer. We then evenly inoculated on brainheart infusion broth agar plates with 50 µL aliquots of the dilution. Paper disks with 5 mm diameter were pasted on the surface of the solid culture medium after high-pressure sterilization. ToAP2A, ToAP2C, and ToAP2D (4/2/1 mg/ml) and the control sample (physiological saline with a concentration of 0.9%) were dripped onto the filter paper with 6 µL, respectively, and cultured in 37°C for 72 h to observe whether there is a zone of inhibition. The diameter of the inhibition zone was measured and recorded with a vernier caliper. The above steps were repeated three times to calculate the average value and standard deviation of the inhibition zone diameter of each sample.

### Minimal Inhibitory Concentration Assay of ToAP2D *In Vitro*

The minimal inhibitory concentration (MIC) of ToAP2D against *S. globosa* was determined using the microdilution assay. In brief, twofold serial dilutions of ToAP2D were prepared in 96-well polystyrene microplates to a final volume of 100 µL. Final concentrations of ToAP2D ranged from 5 mg/ml to 0.62 μg/ml. In each plate, wells were included without peptide as control. Then, 100 µL of the liquid medium (brainheart infusion broth) was added to each well with a final concentration of 1 × 10^8^ cfu/ml for *S. globosa*. The plates were incubated at 37°C for 7 days. In addition, 20 µL of culture from each well was inoculated on the brainheart solid medium and cultured in a constant temperature incubator at 37°C for 7 days to observe the growth of colonies. The MIC was defined as the lowest ToAP2D concentration that completely inhibited visible *S. globosa* growth at the end of the incubation period. The experiments were performed at least three times on separate dates.

### Serum Preparation and ToAP2D Serum Stability Assays

The stability of ToAP2D in human serum was analyzed using the inhibition zone test. 5 ml of healthy human blood (from the physical examination center of our hospital) was extracted using a sterile syringe, stored at room temperature for 1 h, and then stored at 4°C overnight. After 24 h, the blood was centrifuged at 1,000 rpm/min for 10 min, and 50 µL of serum in the supernatant was gently sucked out with a pipette gun. The serum was diluted with 50 µL of 0.9% normal saline, and then, the antimicrobial peptide ToAP2D was dissolved in the diluted serum.

To determine the stability of ToAP2D in human serum, ToAP2D (final concentration 4 mg/ml) was mixed with the diluted serum for 0, 0.5, 1, 2, 4, 6, 8, and 10 h at 37°C, respectively. In brief, *S. globosa* was inoculated on brainheart infusion broth agar plates, 5.0 mm paper disks were placed on top, and 6 µL aliquots of the serumpeptide mixture were added to the paper disks at each incubation time point. After 24 h of incubation, the inhibitory zones against *S. globosa* were measured and recorded.

### ToAP2D Acute Toxicity Analysis

To examine their toxicity, an *in vivo* animal study was performed. The 10 healthy BALB/c mice were randomly divided into ToAP2D group (*n* = 5) and the control group (*n* = 5). The mice in the ToAP2D group were injected with ToAP2D through the tail vein at a dose of 60 mg/kg, and the control group mice were injected with the same volume of normal saline. After 24 h, the major organs (e.g., brain, heart, liver, and kidney) of the mice were excised and fixed in 10% buffered formalin for 1 day. The tissues were then embedded in paraffin and sectioned for hematoxylin and eosin (HE) staining.

### *In Vitro* ToAP2D Treatment Protocol

We inoculated *S. globosa* (1 × 10^8^ cfu/ml) on the brain heart solid culture dish evenly in the clean bench. After 24 h, the 12 culture dishes were randomly divided into control group (n = 6) and the study group (*n* = 6). The *S. globosa* control group was not treated, while the study group was treated with ToAP2D (4 mg/ml). The antifungal capacities of antibacterial peptide ToAP2D against *S. globosa in vitro* were examined 24 h later.

### Observation of *S. globosa* Morphology After ToAP2D Treatment

*S. globosa* in both groups was cultured in 24-well tissue culture plates with cell slide for 4 h. After removing the supernatant and washing each well with PBS three times, we fixed both strains with 2.5% glutaraldehyde for 2 h. After fixation, disks were rinsed three times with PBS and dehydrated in an ethanol series (30, 50, 70, 80, 90% for 7 min each, 100% for 10 min). Disks were dried at conventional critical point and vacuum sputter gold coated. The morphology of *S. globosa* was observed using a scanning electron microscope (SEM) (Hitachi, Tokyo, Japan).

### Cell Apoptosis and Necrosis After ToAP2D Treatment

Cell apoptosis and necrosis were analyzed using the Hoechst 33342–propidium iodide (PI) double-staining apoptosis-detection kit (Beyotime Institute of Biotechnology, Jiangsu, China). The strains of the study and control groups were centrifuged at 3,000 rpm for 5 min and then resuspended at 800 µL staining buffer. The strain solution was rinsed twice with PBS and then diluted to 1 × 10^6^ cfu/ml with a hemocytometer. 5 µL of Hoechst 33342 and 5 µL of PI were incubated into the solution and mixed according to the manufacturer’s instructions. The solution was washed with PBS once after ice bath for 30 min. The smears were observed under a laser scanning confocal microscope (Zeiss LSM 780, German).

### ROS and Mitochondrial Membrane Potential Detection After ToAP2D Treatment

ROS and mitochondrial membrane potential levels were measured using an ROS detection assay kit and a JC-1 kit (Beyotime Institute of Biotechnology, Jiangsu, China), respectively. The strains of the study and control groups were diluted to 1 × 10^8^ cfu/ml.

To detect the ROS level change of *S. globosa* after laser irradiation, the strains in the study and control groups were incubated in dichlorodihydrofluorescein diacetate (DCFH-DA) (10 µM) for 20 min at 37°C. After being washed three times with YPD, *S. globosa* was treated with an active oxygen positive control reagent, an experimental group reagent, and a control group reagent, respectively. After 20 min, the fluorescence intensity was monitored by the microplate fluorescence reader. The ROS level was calculated as the manufacturer’s instructions.

To detect the changes in mitochondrial membrane potential of *S. globosa* after ToAP2D treatment, strains in the study and control groups were stained with JC-1. After being washed three times and resuspended in PBS, *S. globosa* in the study and control groups was added with JC-1 (5 μM) and mixed incubation was performed at 37°C for 20 min. The absorbance was measured with the microplate fluorescence reader at the excitation wavelength of 490 nm and emissions at 530 nm.

### Metacaspase Detection After ToAP2D Treatment

Metacaspases activity was measured using the Cas-pACE FITC-VAD-FMK *in situ* marker (Promega, Fitchburg, WI, United States). The strains of the study and control groups were centrifuged at 3,000 rpm for 5 min and diluted to 1 × 10^6^ cfu/ml. *S. globosa* was washed with PBS once before staining with 10 µM CaspACE FITC-VAD-FMK. After 20 min of incubation at 37°C, *S. globosa* was washed twice with PBS, and the fluorescence absorption was detected by flow cytometry. The excitation light wavelength and the emission light wavelength were set to 494 and 518 nm, respectively.

### Animals Treatment Protocol

The animal protocol was approved by the Institutional Ethics Committee for Animal Use in Research, and our animal care followed Animal Care guidelines of the Jilin University. Mice were randomly divided into study group (*n* = 51) and the healthy control (HC) group (*n* = 6). Mice in the study group were injected with 0.05 ml *S. globosa* suspension (1 × 10^8^ cfu/ml) on their footpads after alcohol cotton ball sterilization. The condition of the skin was monitored daily. On Day 10, we proved that the mouse model was successfully established evidenced by mice histopathology changes (*n* = 3) in the study group. After that, CO_2_ lattice laser was used to promote ToAP2D absorption, and the mice of the study group were further divided into infection group (*n* = 12), laser group (*n* = 12), laser + ToAP2D group (*n* = 12), and itraconazole group (*n* = 12), and the antifungal treatments were given subsequently.

In the laser group, the injection feet were subjected to CO_2_ lattice laser 10,600 nm irradiation at room temperature with an irradiation energy density of 120 mJ/mm^2^. The laser scanning area was 15 mm × 15 mm with a lattice density of 0.3 mm and 25.0% coverage rate. The laser treatment was administered every 3 days on Day 10, 14, 18, and 22. In the laser + ToAP2D group, mice were subjected to CO_2_ lattice laser irradiation with the same parameters in the laser group to assist the absorption of ToAP2D. Almost immediately after laser irradiation, the antibacterial peptide ToAP2D (4 mg/ml, diluted with 0.9% normal saline) was repeatedly rolled onto the laser-irradiated part of the footpad, until it was completely absorbed every day. In the itraconazole group, mice were gavaged with itraconazole at a dose of 60 mg/kg daily. The size of the footpads of mice was measured every 3 days and analyzed using Photoshop. On Day 18 and 26, six mice in each group were sacrificed, and their feet tissues were collected for further skin histology and immunohistochemistry studies.

### Histology

The fungal inoculation sites of mice skin were isolated and fixed with 10% buffered formalin. Samples were dehydrated, paraffin-embedded, and sliced into 5 µm sections. Then, they were stained with HE to observe the inflammation in the tissue. The software ImageJ was used to quantify the percentage area of inflammatory cells on Day 18 and Day 26, respectively.

The expression of caspase-3 was detected using immunohistochemistry to evaluate the apoptosis in mice footpad tissues after different treatments. In brief, paraffin-embedded sections of mice footpad tissue in different groups were dewaxed and gradually rehydrated before being immersed in an EDTA solution (pH 9.0). After 150 s of heating at 120°C, the antigen was slowly cooled to room temperature. After fixation, immunostaining blocking solution was used to block and incubate with primary antibody at 4°C overnight. The caspase-3 rabbit polyclonal antibody (AF0081, Beyotime Biotechnology; 1:100) was added. After washing with PBS, the tissue sections were incubated with an HRP-conjugated secondary antibody for 20 min at 37°C and visualized with diaminobenzidine (DAB), followed by counterstaining with hematoxylin, hydrochloric acid ethanol differentiation, phosphate buffer solution back blue, gradient ethanol dehydration, xylene transparent, and neutral gum seal. The relative expression of apoptotic factor caspase-3 was assessed by average optic density (AOD) obtained from ImageJ, AOD = integrated optical density (IOD)/area.

### Statistical Analysis

Graphpad Prism 7.0 software (Graphpad Software, La, Jolla, CA, United States) was used to conduct the statistical analysis and figure production. Data were summarized as median and range for non-normal distribution data or mean ± standard deviation (SD) for normally distributed data. The quantitative analysis of caspase-3 was performed to assess the average optic density (AOD) obtained from ImageJ. Statistical significance was determined using the one-way analysis of variance (ANOVA), Mann–Whitney *U* test, or Student’s t-test. All *in vitro* study experiments were performed in triplicate. *p* < 0.05 was considered to be statistically significant.

## Results and Discussion

### Peptide Design and Functional Screening

The sequences and bioinformatic characterization of the ToAP2 and its derived peptides ToAP2A, ToAP2B, ToAP2C, and ToAP2D are presented in Table 1, 2.

**TABLE 1:** The comparison of ToAP2 amino acid sequences and derived peptides.

Peptide	Sequence
ToAP2	FFGTLFKLGSKLIPGVMKLFSKKKER
ToAP2A	GSKLIPGVMKLFSKK
ToAP2B	FFGTLFKLGSKLIPGV
ToAP2C	GAKLIPGVMKLFRKK
ToAP2D	GDKLIPGVMKLFRKK

**TABLE 2:** The comparison of physical and chemical properties of ToAP2 and its derived peptides.

Peptide	Molecular weight (Da)	Sequence length	alpha-Helix(%)	PI	Net charge	GRAVY	Total hydrophobic ratio (%)	Aliphatic index	Instability index	Boman index (kcal/mol)
ToAP2	3,000.73	26	69.23	10.68	+6	0.019	42	86.15	−10.33	0.66
ToAP2A	1,633.07	15	60.00	10.48	+4	0.093	40	97.33	−3.91	0.18
ToAP2B	1724.12	16	37.50	10.00	+2	1.025	50	115.62	−17.74	-1.15
ToAP2C	1,686.18	15	66.67	11.33	+5	0.02	46	104	−9.57	0.62
ToAP2D	1730.19	15	66.67	10.46	+4	-0.333	40	97.33	−9.57	1.32

A previous study reported that antimicrobial peptide ToAP2 (AP02759), a 26-amino acid peptide, showed high antifungal and antifungal biofilm activity but low hemolytic and cytotoxic activity, indicating that this peptide has great potential for the development of new antifungal drugs (Guilhelmelli et al., 2016). Based on ToAP2, a series of peptides were designed by incorporating effective fragments and amino acid substitutions.

Modifying the antibacterial peptide structure, such as sequence length and special amino acids, can improve its antifungal activity and reduce toxicity (Lima et al., 2021; Schafer et al., 2021). To test the hypothesis, we first designed ToAP2A and ToAP2B by integrating effective fragments. The bioinformatic characterization data indicated that the sequence length and molecular weight of ToAP2A and ToAP2B were much shorter than that of ToAP2, while the hydropathic and aliphatic index increased. Meanwhile, ToAP2A exhibited enhanced capacity in alpha-helix, instability index, and protein binding ability than ToAP2B. Collectively, ToAP2A has superior properties than ToAP2B and ToAP2.

ToAP2A was used for further modification through amino acid substitution. ToAP2C was first designed by substituting Ser-2/14 residues with Ala-2 and Arg-14. Though ToAP2C exhibited improvements in the positive net charge, alpha-helix, aliphatic index, and protein binding ability, ToAP2A displayed higher hydrophilicity and stability. We further investigated the effect of Ser substitution with Asp-2 and Arg-14 on the property of ToAP2A derivative. The resulting peptide ToAP2D showed better performance in alpha-helix and protein binding ability than ToAP2A.

Taken together, all these results indicated that the properties of ToAP2A, ToAP2C, and ToAP2D are improved compared with ToAP2B and ToAP2, especially in molecular weight, sequence length, and stability.

### Antifungal Activity, Serum Stability, and Acute Toxicity Assays of Antimicrobial Peptides

**FIGURE 1:**
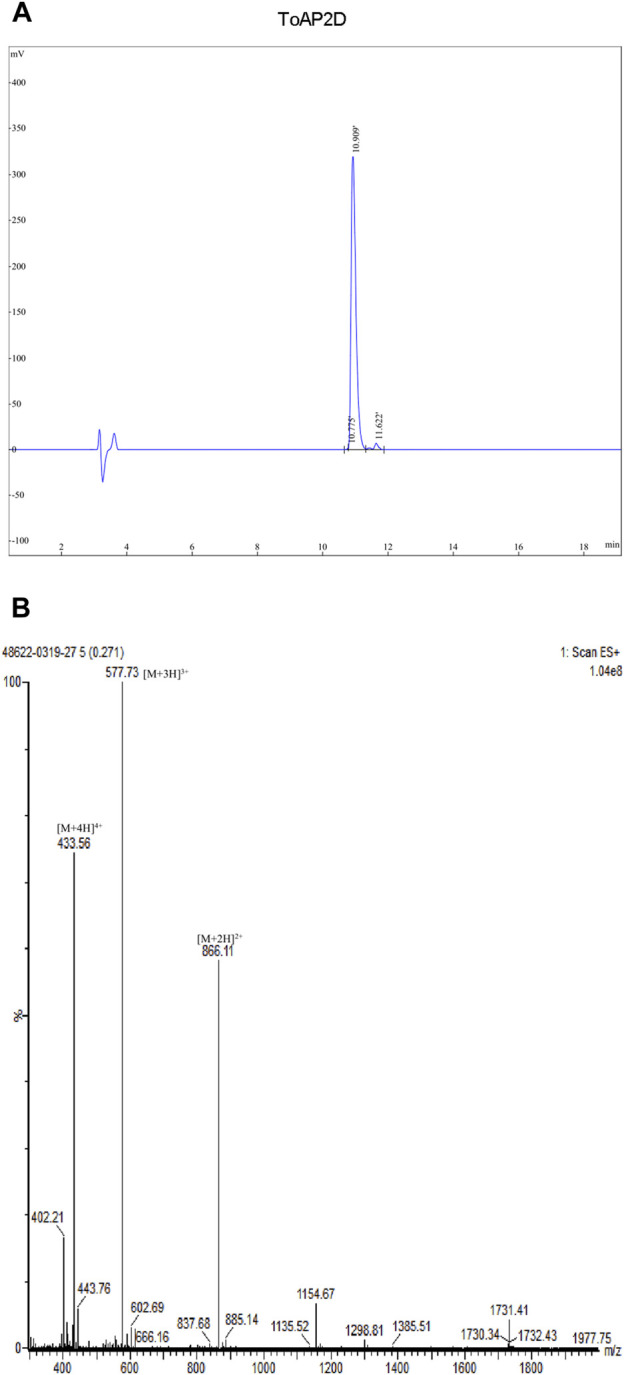
Synthesized ToAP2D purity was determined using reversed-phase high-performance liquid chromatography (RP-HPLC) and electrospray ionization mass spectrometry (ESI-MS). **(A)** RP-HPLC analysis **(B)** ESI-MS analysis.

Antimicrobial peptides ToAP2A, ToAP2C, and ToAP2D were synthesized, and their purities (>95%) were determined using reversed-phase high-performance liquid chromatography (RP-HPLC) and electrospray ionization mass spectrometry (ESI-MS) (Figure 1 and Fig. S1–S4).

The inhibition zone test was employed to analyze the ToAP2D antifungal activities (Zhang et al., 2021). The results showed that three peptides had no inhibition zone at the concentration of 1 mg/ml. When the concentration increased to 2 mg/ml and 4 mg/ml, they both showed antifungal activities against *S. globosa*, and the noticeable inhibition zone diameter was at 4 mg/ml. At the same concentration (4 mg/ml), the inhibition zone average diameter of ToAP2D (11.56 ± 0.36 mm) was significantly greater than that of ToAP2A (9.23 ± 0.34 mm) and ToAP2C (8.12 ± 0.33 mm), suggesting that ToAP2D has the strongest antifungal activity against *S. globosa* (Table 3). We further determined that the minimal inhibitory concentration (MIC) of the ToAP2D against *S. globosa* by the microdilution assay was 156.25 μg/ml.

**TABLE 3:** Antibacterial diameter of antimicrobial peptides *in vitro*.

group	concentration (mg/ml)		
	4	2	1
	Inhibition zone (mm) mean ± standard deviation (SD)		
ToAP2A	9.23 ± 0.34^***^	6.59 ± 0.34^***^	5.00 ± 0.00
ToAP2C	8.12 ± 0.33^***^	6.37 ± 0.27^***^	5.00 ± 0.00
ToAP2D	11.56 ± 0.36^***^	6.86 ± 0.25^***^	5.00 ± 0.00
control sample	5.00 ± 0.00	5.00 ± 0.00	5.00 ± 0.00

Normally distributed data are shown as the mean ± SD.

* mean compared to control sample, *p* < 0.05.

** mean compared to control sample, *p* < 0.01.

*** mean compared to control sample, *p* < 0.001.

**FIGURE 2:**
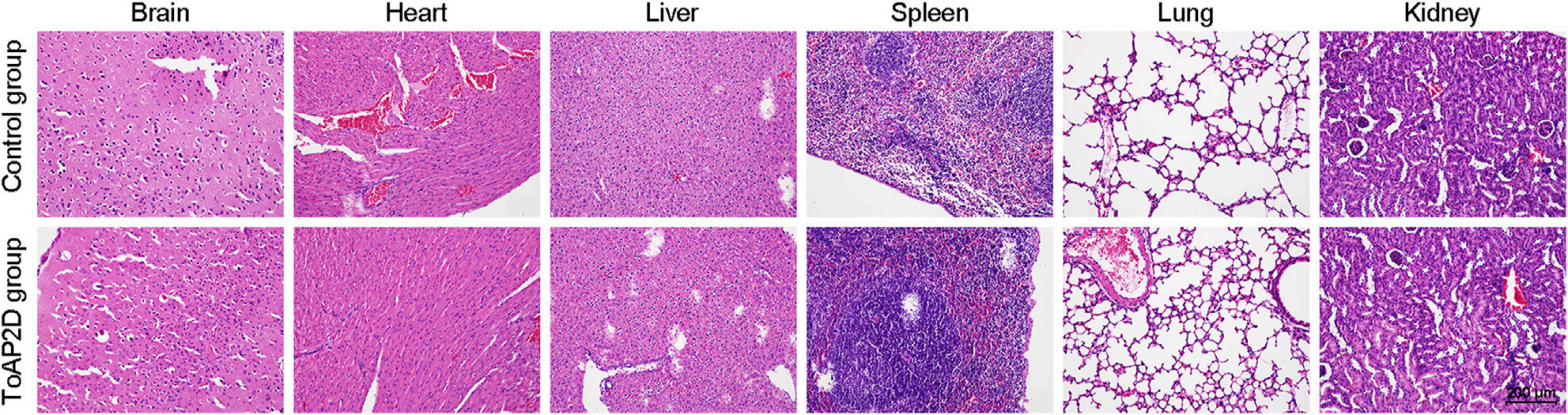
HE staining of mice major organs (brain, heart, liver, spleen, lung, and kidney) in the ToAP2D treatment and control group. The scale bar = 200 μm.

The serum stability of the drug determines whether it can be administered intravenously (Mwangi et al., 2019). To examine its administration potential, we tested the stability of ToAP2D in human serum. After 10 h of incubation in serum, ToAP2D (4 mg/ml) showed that its antifungal activity against *S. globosa* was comparable to the original drug (>90%). Given that the ToAP2D sequence only contains 15 amino acid residues, it is difficult for ToAP2D to be inactivated by the enzymes in serum. The results suggested that the antibacterial peptide ToAP2D is stable in serum, which may lay a good foundation for the development of anti-bacterial drugs. Additionally, the acute toxicity for the ToAP2D was evaluated by HE staining of the main organs of mice (Li et al., 2020). As shown in Figure 2, no ultrastructural changes were observed in all main organs of the control and ToAP2D treated groups, which suggested that ToAP2D has no acute toxicity.

Overall, these results indicated that the ToAP2D has great potential for the treatment of *S. globosa* infection with serum stability and no acute toxicity.

### *S. globosa* Morphological Changes After ToAP2D Treatment *In Vitro*

**FIGURE 3:**
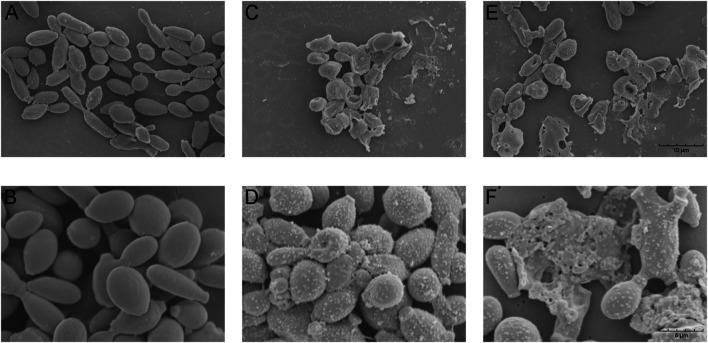
Effect of ToAP2D treatment on morphological changes of *S. globosa* was observed by SEM **(A,B)** *S. globosa* in the control group was normal morphology. **(C–F)** *S. globosa* morphological changes after ToAP2D treatment. The scale bars in **(A)**, **(C)**, and **(E)** represent 10 μm, and those in **(B)**, **(D)**, and **(F)** represent 5 μm.

Antimicrobial peptides often induce fungal morphology and structure changes (Vieira Bard et al., 2014; Kong et al., 2019). To assess the possible mechanism of ToAP2D action, the morphology of *S. globosa* was observed using a scanning electron microscope (SEM). *S. globosa* showed normal morphology in the control group, which has a regular oval shape, complete and smooth surface, clear borders with no wrinkles (Figures 3A,B). However, we found that the *S. globosa* morphology changed significantly after ToAP2D treatment. There are many granular or vesicle-like structures that appeared on the surface, and some areas showed irregular shapes (e.g., depressions, holes, and leakage of contents) (Figures 3C–F). This indicated that ToAP2D treatment may destroy the cell wall and membrane of *S. globosa*, thereby selectively destroying its permeability, reducing the intracellular osmotic pressure, causing a large amount of cytoplasm to leak out, and promoting its death. In the meantime, these results indicated that ToAP2D might have a necrosis effect on *S. globosa*. Additionally, histology research showed that morphological changes, including cell shrinkage, nuclear karyorrhexis, and plasma membrane blebbing phenomenon, are also involved in the process of apoptosis (Saikumar and Kar, 2008) (Coleman and Olson, 2002).

### *S. globosa* Apoptosis After ToAP2D Treatment *In Vitro*

**FIGURE 4:**
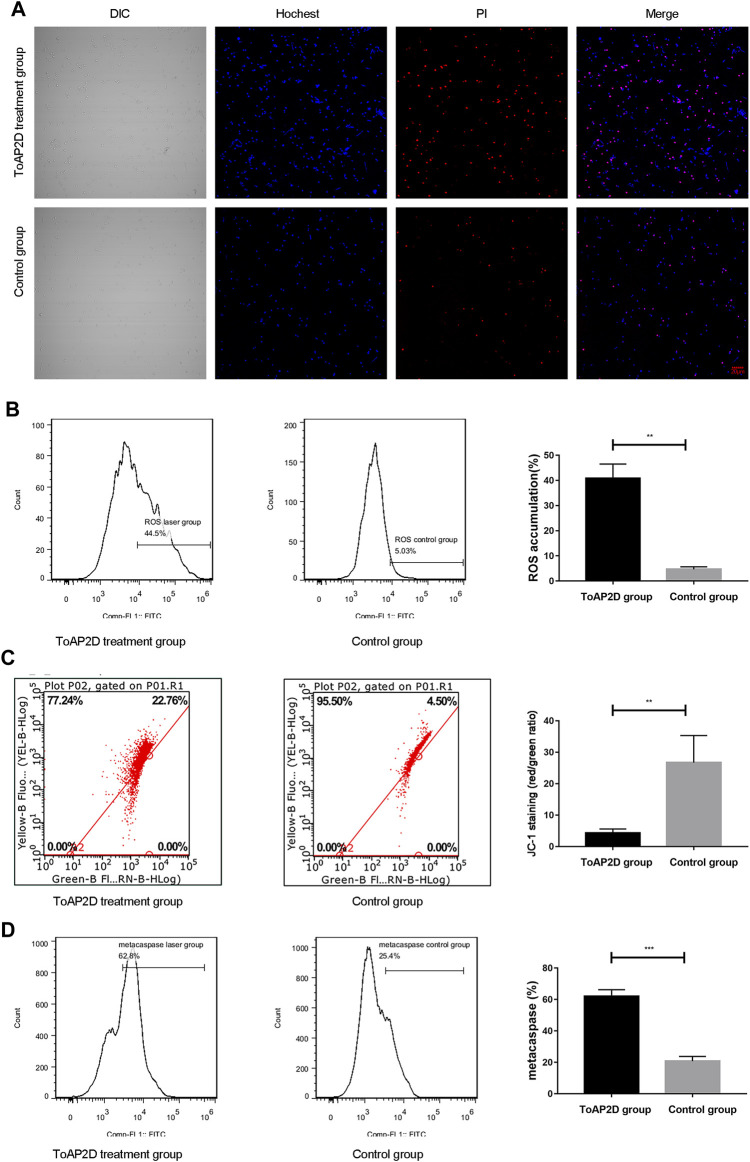
Effect of ToAP2D treatment on apoptosis of *S. globosa*. **(A)** Laser scanning confocal microscope was used to observe the apoptosis and necrosis effect of ToAP2D treatment. The scale bar = 20 μm. **(B)** Flow cytometry was used to detect the ROS accumulation. **(C)** Flow cytometry was used to detect the mitochondrial membrane potential. **(D)** Flow cytometry was used to detect the metacaspase activation. *p < 0.05, **p < 0.01, and ***p < 0.001 indicate statistical significance.

Apoptosis and necrosis are the two major forms of cell death, which are crucial for maintaining the body development and removing abnormal cells (Ju et al., 2021; Riwaldt et al., 2021). To investigate the effect of ToAP2D on apoptosis and necrosis, a Hoechst/PI double staining was performed. We observed that there was only weak blue fluorescence and red fluorescence in the control group, indicating that *S. globosa* was in a normal state. However, strong blue fluorescence and red fluorescence (Hoechst+/PI+) were observed in the laser irradiation group (Figure 4A), suggesting that ToAP2D treatment significantly promoted the apoptosis and necrosis of *S. globosa*.

Great efforts have been made to explore the mechanism of *S. globosa* apoptosis. ROS is an important regulator of cell apoptosis, which can damage DNA and affect cell cycle process (Ikner and Shiozaki, 2005). After ToAP2D treatment, we found that the ROS accumulation was significantly elevated (40.84 ± 5.69% versus 4.65 ± 1.00%; *p* = 0.004) (Figure 4B), indicating that ROS-mediated apoptosis pathway was activated.

The decrease in mitochondrial membrane potential signifies cell apoptosis (Guaragnella et al., 2012). A recent study revealed that ROS promoted the irreversible over-opening of mitochondrial permeability transition pores, leading to the decrease in mitochondrial membrane potential (Cho and Lee, 2011). We thereby investigated the mitochondrial membrane potential between ToAP2D and control groups. Our results showed that ToAP2D treatment reduced the mitochondrial membrane potential (4.30 ± 1.29 versus 26.73 ± 8.58; *p* = 0.004) (Figure 4C), indicating that ToAP2D caused mitochondrial dysfunction.

Metacaspases are orthologues of caspases, which are crucial mediators of apoptosis in fungi and plants. ROS generation can induce caspase-dependent apoptosis (Madeo et al., 2009; Qi et al., 2019). After ToAP2D treatment, the metacaspase activation of the study and control groups reached 62.05 ± 4.15% and 20.73 ± 3.05%, respectively (*p* < 0.001) (Figure 4D).

Taken together, these results indicated that the ToAP2D treatment resulted in dysfunctional mitochondria and ROS accumulation, and activated the caspase-dependent apoptosis pathway, which triggered *S. globosa* apoptosis.

### *In Vivo* Skin Infection

CO_2_ lattice laser has strong penetrability, which can precisely control the depth of skin penetration and punctate exfoliation (Wenande et al., 2017). Additionally, CO_2_ laser surgery is less invasive and causes reduced trauma and pain (Artzi et al., 2019). Recent studies indicated that its thermal effect can inhibit fungi, initiate local immune responses, and assist the absorption of drugs (Orringer et al., 2010; Sobhi et al., 2020). In our study, we investigated the efficacy and mechanism of ToAP2D against *S. globosa*, and CO_2_ lattice laser was used as a way to promote its absorption. Itraconazole is the first-line antifungal agent for sporotrichosis (García A. Carnero et al., 2018; Abbotsford et al., 2021), and it was used to evaluate the ToAP2D anti-fungal activity.

**FIGURE 5:**
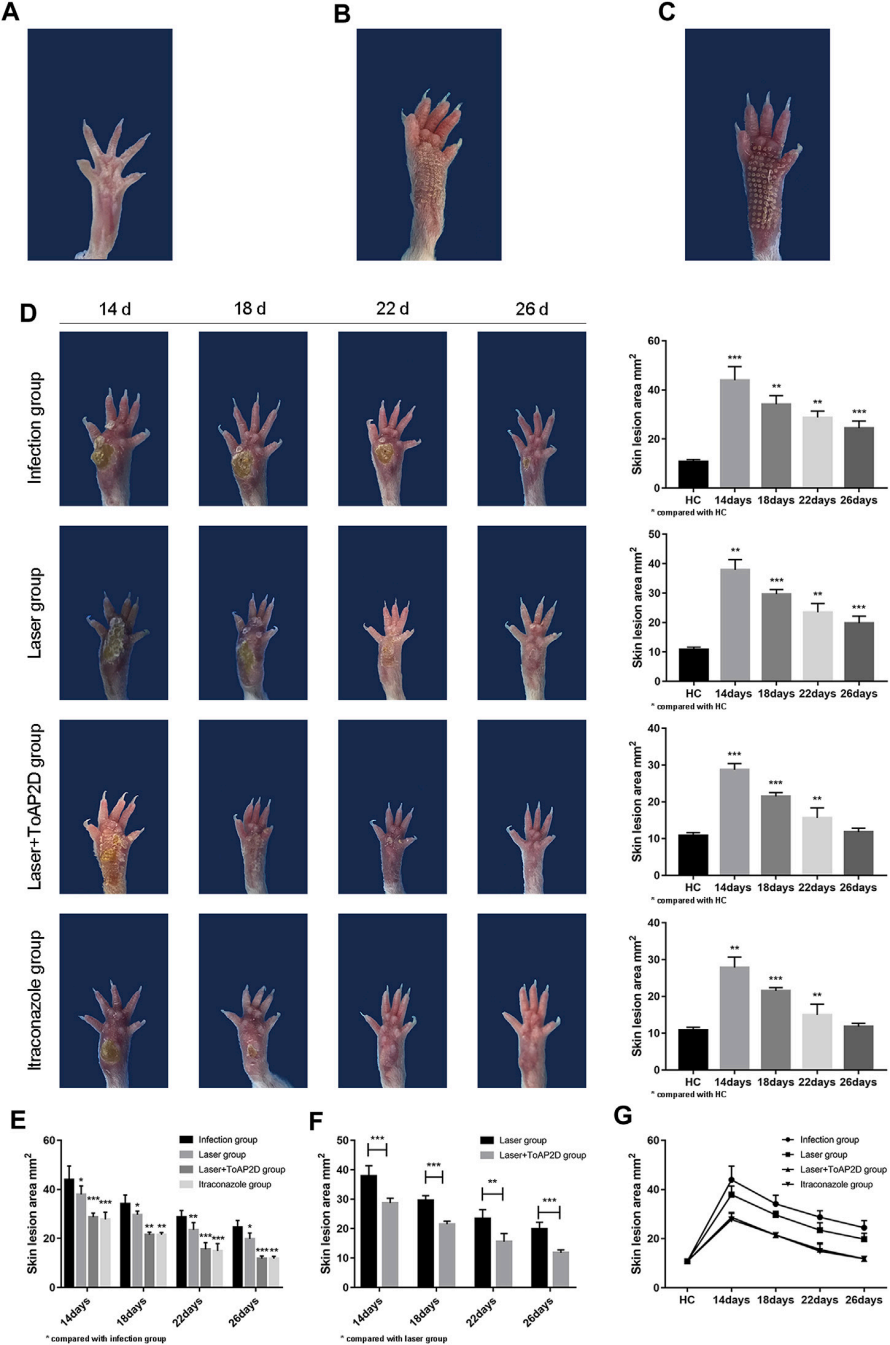
The mice skin lesion area comparison after different treatments. **(A)** HC group. **(B)** Laser treatment. **(C)** Laser + ToAP2D treatment. **(D)** Compared with the HC group, the footpads’ size after different treatments from Day14 to 26. **(E)** Compared with the infection group, the footpads’ size in the laser, laser + ToAP2D, and itraconazole group from Day14 to 26. **(F)** Compared with the laser group, the footpads’ size in laser + ToAP2D group from Day14 to 26 **(G)** Analysis on the trend of the skin lesion area of all groups mice followed up time from Day14 to 26. *p < 0.05, **p < 0.01, and ***p < 0.001 indicate statistical significance.

The footpad sizes of the mice were measured on Day 14, 18, 22, and 26 after *S. globosa* injection (Table 4), respectively. The size of mouse skin lesion area was 10.83 ± 0.84 mm^2^ in the HC group (Figure 5A). The mouse footpads after laser and laser + ToAP2D treatment are distinct from the healthy one (Figures 5B,C). The size of footpads in laser + ToAP2D and itraconazole groups almost became normal on Day 26, which were both faster than mice in the infection and laser groups (Figure 5D). From Day 14 to Day 26, a significant size decrease in mouse footpads was observed in all treatment group compared with the infection group (Figure 5E), while the treatment efficiency in the laser + ToAP2D group was better than that of the laser-only group (Figure 5F). Meanwhile, no significant difference was observed between the laser + ToAP2D and itraconazole groups (Figure 5G), suggesting that their anti-fungal effects were almost the same.

**TABLE 4:** The mice foot skin lesion area analysis.

Parameters	Study group	Day14	Day18	Day22	Day26
		(n = 6)	(n = 6)	(n = 6)	(n = 6)
Skin lesion area (mm^2^)	Infection group	43.99 ± 5.56***	34.19 ± 3.53**	28.77 ± 2.61**	24.48 ± 2.90***
	Laser group	37.91 ± 3.50**	29.65 ± 1.54***	23.48 ± 3.00**	19.81 ± 2.39***
	Laser + ToAP2D group	28.71 ± 1.66***	21.49 ± 1.03***	15.62 ± 2.73**	11.81 ± 1.02
	Itraconazole group	27.82 ± 2.84**	21.47 ± 0.92***	14.94 ± 2.93**	11.81 ± 0.85

Normally distributed data are shown as the mean ± SD.

* mean compared to HC, *p* < 0.05.

** mean compared to HC, *p* < 0.01.

*** mean compared to HC, *p* < 0.001.

### Histopathology and Immunohistochemistry (IHC)

**FIGURE 6:**
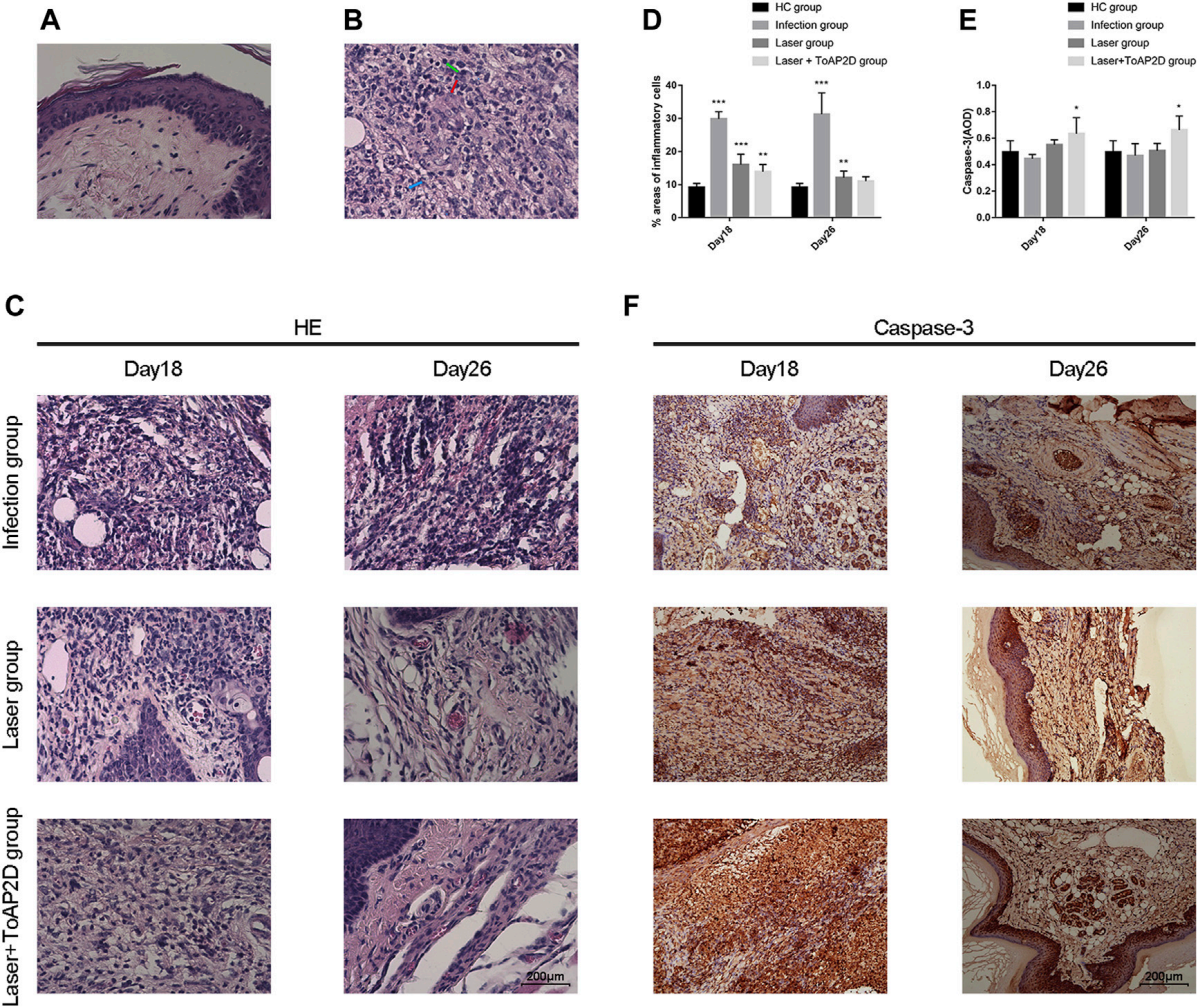
The mice histopathology and IHC comparison after different treatments. We compared the histopathological changes and caspase-3 expression of mice skin tissues on Day18 and 26. **(A)** HC group. **(B)** Study group on Day 10 (red arrow: epithelioid cell, green arrow: mononuclear cell, blue arrow: neutrophil). **(C)** Histopathological changes of mice skin tissues after different treatments on Day 18 and 26. **(D)** Compared with HC, the percentage area of inflammatory cells in different treatment groups on Day 18 and 26, respectively. **(E–F)** Compared with HC, the IHC expression of caspase-3 in different treatment groups on Day 18 and 26, respectively.

Recent studies revealed that inflammation is closely correlated with the host resistance to sporotrichosis (Batista-Duharte et al., 2018; García-Lozano L. C. et al., 2018). Histopathological analysis is a common method to evaluate the inflammatory response, which was used in this study to assess the ToAP2D treatment efficacy. The normal histopathology of the mice is shown in Figure 6A. After 10 days of infection, three mice in the study group were randomly taken for histopathological examination. We observed suppurative inflammation with epithelioid cells, mononuclear cells, and neutrophils formation, confirming that the *S. globosa* infection mouse model was established successfully (Figure 6B).

The histopathological changes of the mouse footpads in different groups were further analyzed on Day 18 and 26 (Figure 6C). On Day 18, suppurative granulomas with numerous neutrophils and lymphocytes were observed in the infection group, while the inflammation was significantly less in the laser and laser + ToAP2D groups. On Day 26, though inflammation still existed in the infection and laser group, the number of inflammatory cells in the laser group was significantly lower than those in the infection group. In comparison, the mouse histopathology in the laser + ToAP2D group almost returned to normal, in which the pus-like inflammatory focus was almost disappeared. In addition, quantitative analysis was performed to evaluate the percentage of inflammatory cells on Day 18 and 26. The laser + ToAP2D group showed the greatest decrease in the area containing inflammatory cells, while a large number of inflammatory cells still existed in the infected group on Day 26 (Figure 6D). Taken together, we demonstrated that ToAP2D promoted mouse footpads recovery, which may be used as an alternative for clinical *S. globosa* treatment.

*In vitro* study, we have shown that antibacterial peptide ToAP2D can promote *S. globosa* apoptosis through dysfunctional mitochondria, ROS accumulation, and metacaspase activation. Caspase-3 is a critical executioner of apoptosis (apotosis-related proteins in cervical intraepithelial neoplasia and squamous cell carcinoma of the cervix). *In vivo* study, we further measured the expression of caspase-3 of mice food pad tissues in different groups. Compared with the HC group (0.50 ± 0.08), our data showed that caspase-3 levels were upregulated in the laser + ToAP2D group on Day 18 (0.63 ± 0.12; *p* = 0.044) and 26 (0.66 ± 0.11; *p* = 0.013), whereas no significant difference was found in the infection and laser groups, indicating that apoptosis was also activated by ToAP2D treatment *in vivo* (Figures 6E,F).

## Conclusion

In this study, we developed three ToAP2 derived peptides with antimicrobial activities. Among them, ToAP2D has the best anti-*S. globosa* efficacy, and its serum stability was good without acute toxicity. ToAP2D showed a robust therapeutic effect on *S. globosa*. ToAP2D inhibited the growth of *S. globosa*. Meanwhile, it also triggered apoptotic pathway in *S. globosa*, including dysfunctional mitochondria, ROS accumulation, and metacaspase activation. In the *in vivo* study, we further demonstrated that ToAP2D inhibited *S. globosa* infection in mice footpads, and the efficacy was nearly equal to that of itraconazole. Moreover, ToAP2D treatment upregulated apoptosis-related protein caspase-3. Collectively, our antimicrobial peptide ToAP2 can inhibit the growth of *S. globosa* and trigger its apoptosis, which may be a potential drug for future sporotrichosis treatment.

## Data Availability

The raw data supporting the conclusions of this article will be made available by the authors, without undue reservation.
