# Supplementary figures and images for: Antifungal Activity of ToAP2D Peptide Against Sporothrix globosa
Source: Front Bioeng Biotechnol. 2021 Oct 21;9:761518. doi: 10.3389/fbioe.2021.761518 (PMC8566951; doi:10.3389/fbioe.2021.761518)

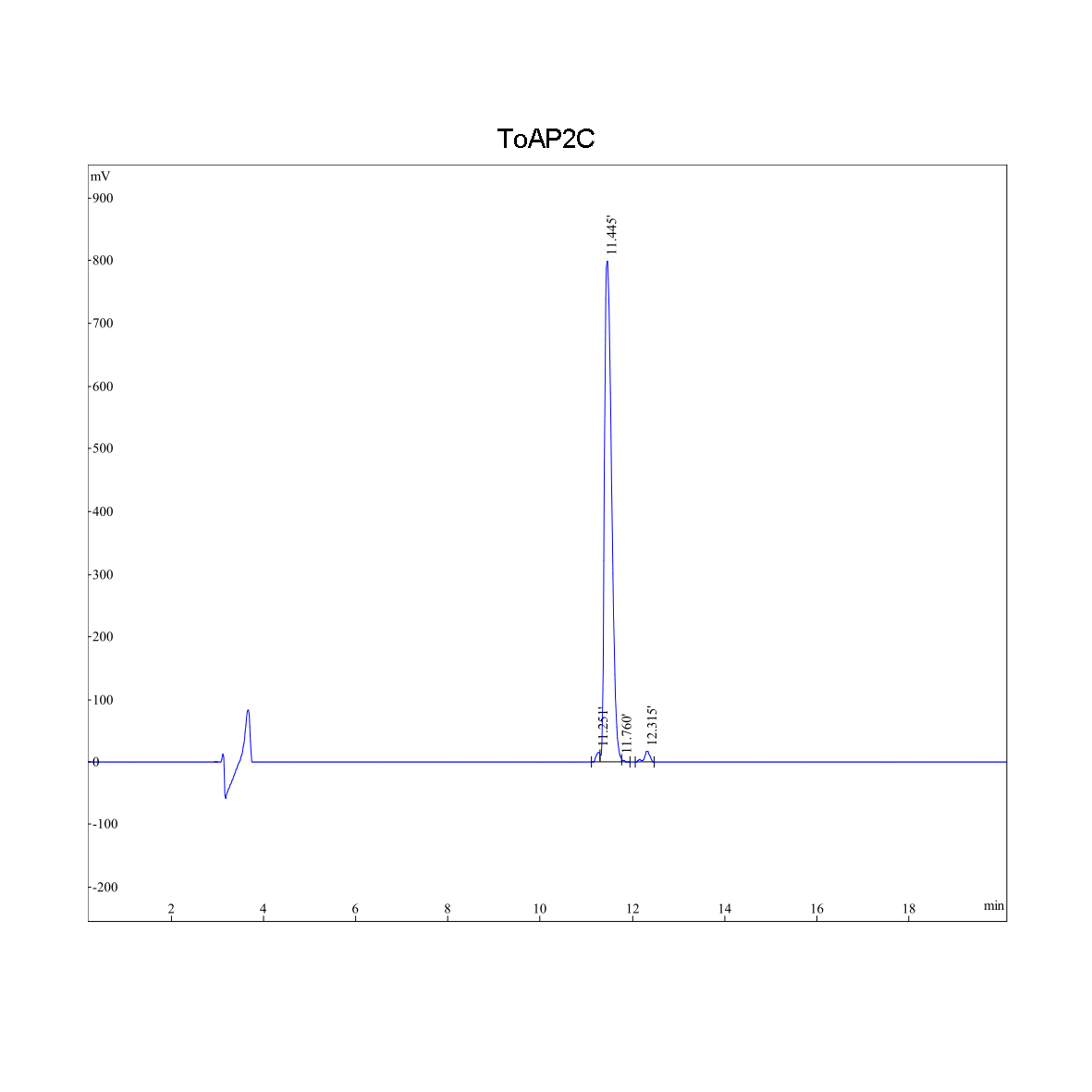

Supplement: Supplementary file 1 [file Image3.TIF]

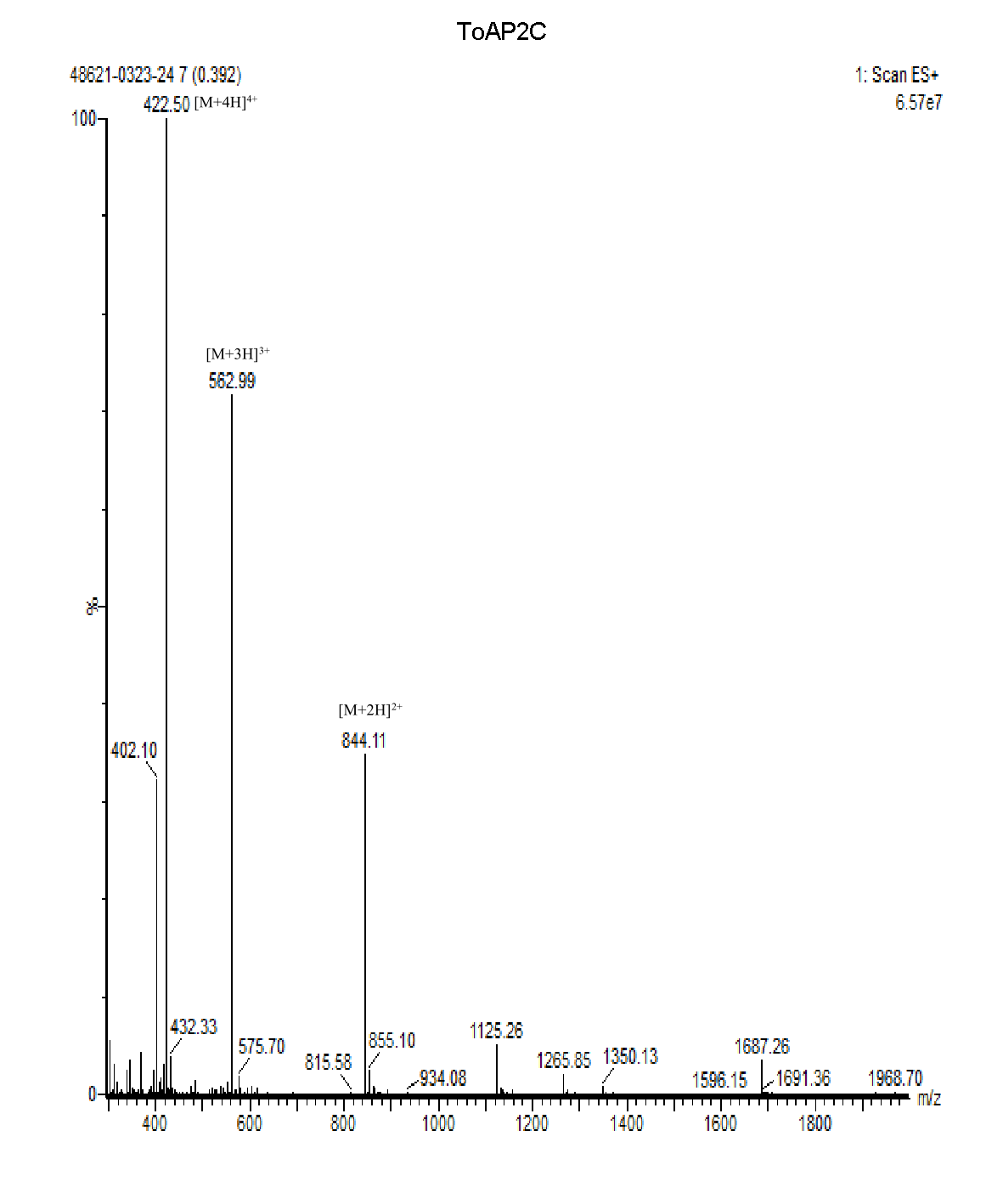

Supplement: Supplementary file 2 [file Image4.TIF]

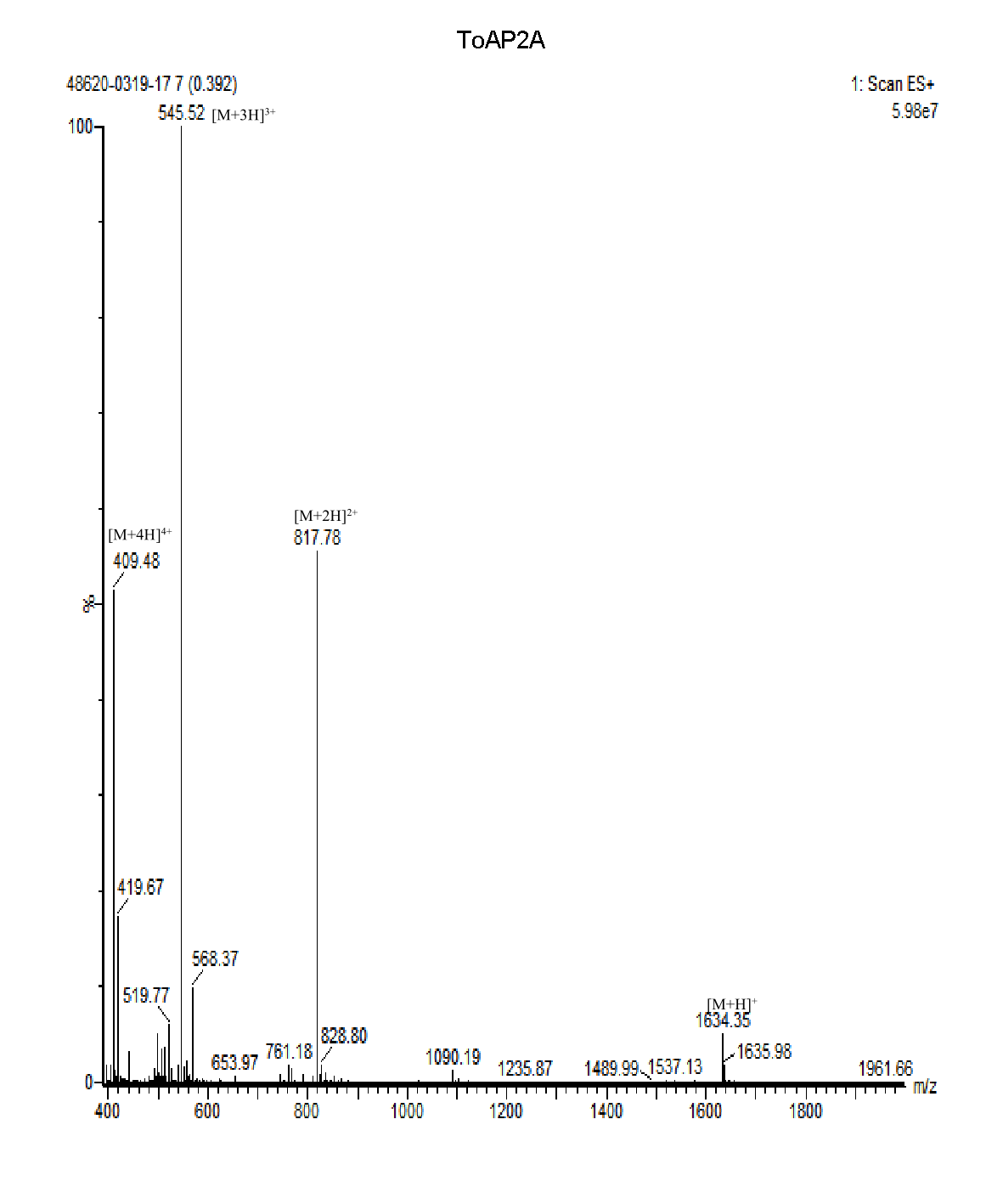

Supplement: Supplementary file 3 [file Image2.TIF]

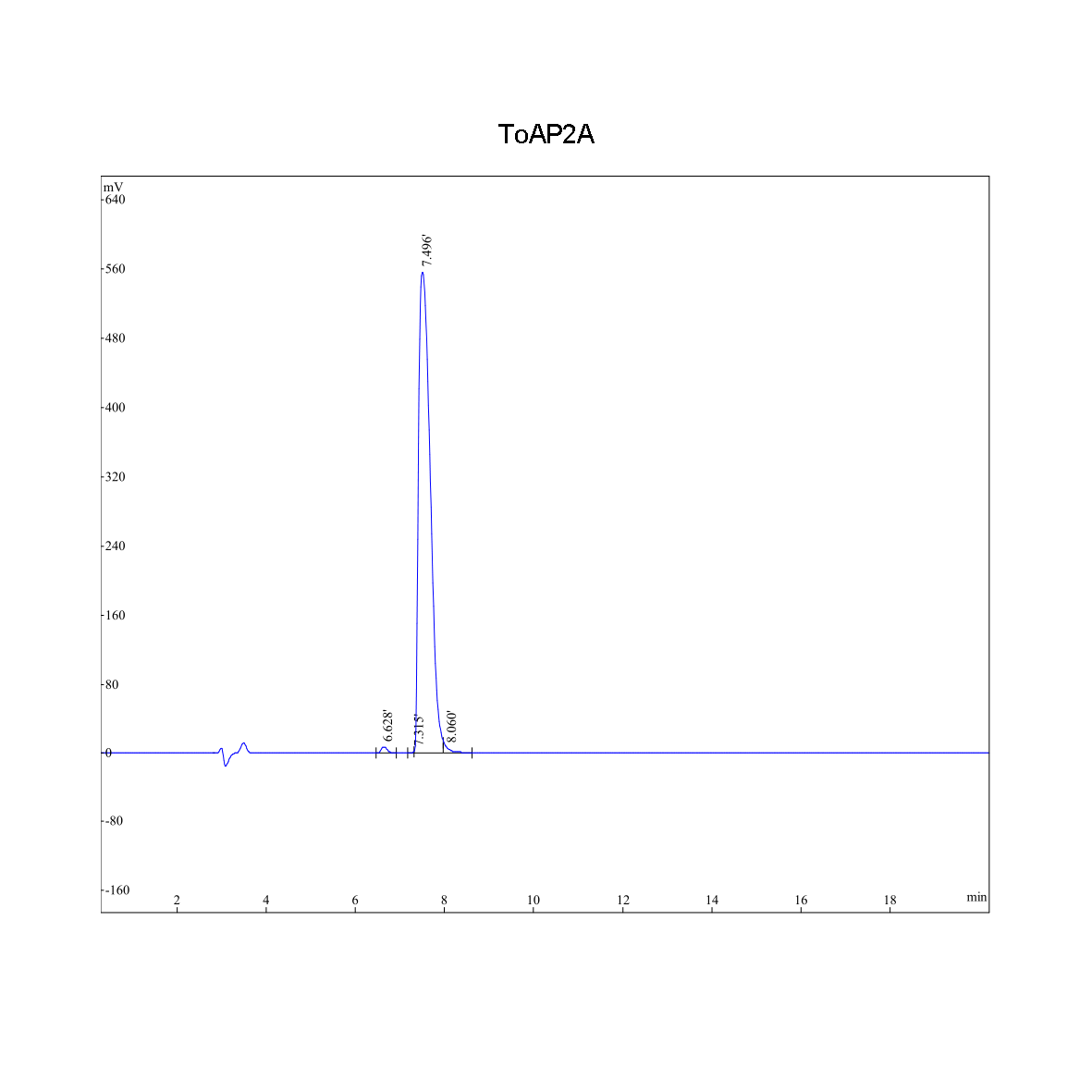

Supplement: Supplementary file 4 [file Image1.TIF]
