# Supplementary material for: Antifungal Activity of ToAP2D Peptide Against Sporothrix globosa
Source: Front Bioeng Biotechnol. 2021 Oct 21;9:761518. doi: 10.3389/fbioe.2021.761518 (PMC8566951; doi:10.3389/fbioe.2021.761518)
Supplement: Supplementary file 5 [file DataSheet1.docx]

Supplementary Material

# Supplementary Figures

**Supplementary Figure 1.** Reversed-phase high-performance liquid chromatography (RP-HPLC) analysis of synthesized antimicrobial peptide ToAP2A purity.

**Supplementary Figure 2.** Electrospray ionization mass spectrometry( ESI-MS) analysis of **s**ynthesized antimicrobial peptide ToAP2A purity.

**Supplementary Figure 3.** RP-HPLC analysis of synthesized antimicrobial peptide ToAP2C purity.

**Supplementary Figure 4.** ESI-MS analysis of **s**ynthesized antimicrobial peptide ToAP2C purity.
